# Optimising secondary prevention and cardiovascular care across Europe: A UK perspective on a common goal

**DOI:** 10.1177/1474515120928279

**Published:** 2020-06-09

**Authors:** Rani Khatib, Jan Keenan

**Affiliations:** 1 Medicines Management and Pharmacy Services, Leeds Teaching Hospitals NHS Trust, Leeds, UK; 2 Leeds Institute for Cardiovascular and Metabolic Medicine, University of Leeds, UK; 3 Oxford Heart Centre, Oxford University Hospitals NHS Foundation Trust, UK

We read with great interest a recent article entitled ‘Secondary prevention and cardiovascular care across Europe: a survey of European Society of Cardiology members’ views’.^1^ In this survey of 479 healthcare professionals from eight countries, the main barriers identified were lack of available cardiac rehabilitation programmes and long-term follow-up, patients’ disease perception and professional attitudes towards prevention. Barriers to prevention varied based on the survey participants’ country of origin; however, there was consensus across all countries on the three most important strategies to improve prevention, namely multidisciplinary interventions, patient education and introducing performance measures.^1^ In the UK, shortcomings in the delivery of secondary prevention programmes have also been linked with suboptimal outcomes for patients with cardiovascular disease (CVD), and recently a group of multidisciplinary team (MDT) members working in secondary prevention in primary and secondary care settings has developed a UK consensus on optimising CVD secondary prevention care.^2^

As co-authors of the UK consensus statement, we were mindful that there is not a uniform, one-size-fits-all model of CVD secondary prevention care, and that within our National Health Service (NHS), the roles undertaken by different MDT members can vary depending on the skillsets and healthcare resources available locally. Within the typical management pathway for post-myocardial infarction follow-up, numerous opportunities for MDT members to deliver secondary prevention care from the time of initial presentation and hospitalisation through to long-term post-discharge follow-up were identified and outlined. In addition, a range of best practice models from across the UK that are currently achieving success in reducing cardiovascular risk were described and endorsed with a view to being adopted or adapted elsewhere. These included a pharmacist-led, hospital-based post-myocardial infarction medicines optimisation programme,^3^ a nurse-led integrated community-based CVD prevention programme and a GP-led one-stop heart failure diagnosis and management clinic model for primary care. All of these models provide practical examples of ways that different healthcare professionals involved in delivering MDT care can work together to improve patients’ outcomes in CVD, for example through recognising and addressing poor adherence to cardioprotective medication.^4^,^5^

The data analysis from Fitzsimons et al.^1^ underlines that secondary prevention is often suboptimal across the European countries included in the European Society of Cardiology (ESC) survey. Given that MDT members are central to delivering CVD secondary care, it is understandable that an Association of Cardiovascular Nursing and Allied Professions (ACNAP) task force is currently examining ways to support greater collaboration between allied professions and to address uncertainties about the roles that different MDT members can play in CVD secondary prevention. We suggest that the UK consensus statement (which is available online as an open-access publication) may provide insights and practical guidance which are of interest to cardiovascular nurses and MDT members working in CVD secondary care across Europe.
